# Association of metabolites of benzene and toluene with lipid profiles in Korean adults: Korean National Environmental Health Survey (2015–2017)

**DOI:** 10.1186/s12889-022-14319-x

**Published:** 2022-10-14

**Authors:** Soon Su Shin, Eun Hye Yang, Hyo Choon Lee, Seong Ho Moon, Jae-Hong Ryoo

**Affiliations:** 1 Department of Preventive Medicine, Graduate School, Kyung Hee University, Seoul, Republic of Korea; 2 Department of Occupational and Environmental Medicine, Kyung Hee University Hospital, Seoul, Republic of Korea; 3 Department of Occupational and Environmental Medicine, School of Medicine, Kyung Hee University, Seoul, Republic of Korea

**Keywords:** Benzene, Toluene, Dyslipidemia, Hypertriglyceridemia, Lipid profiles, Korean National Environmental Health Survey (KoNEHS)

## Abstract

**Background:**

Environmental exposure to benzene and toluene is a suspected risk factor for metabolic disorders among the general adult population. However, the effects of benzene and toluene on blood lipid profiles remain unclear. In this study, we investigated the association between urinary blood lipid profiles and metabolites of benzene and toluene in Korean adults.

**Methods:**

We analyzed the data of 3,423 adults from the Korean National Environmental Health Survey Cycle 3 (2015–2017). We used urinary trans,trans-muconic acid (ttMA) as a biomarker of benzene exposure, and urinary benzylmercapturic acid (BMA) as an indicator of toluene exposure. Multivariate logistic regression analyses were performed to explore the association between blood lipid profiles and urinary metabolites of benzene and toluene. Additionally, we examined the linear relationship and urinary metabolites of benzene and toluene between lipoprotein ratios using multivariate regression analyses.

**Results:**

After adjusting for covariates, the fourth quartile (Q4) of ttMA [odds ratio (OR) (95% confidence interval, CI = 1.599 (1.231, 2.077)] and Q3 of BMA [OR (95% CI) = 1.579 (1.129, 2.208)] were associated with an increased risk of hypertriglyceridemia. However, the Q4 of urinary ttMA [OR (95% CI) = 0.654 (0.446, 0.961)] and Q3 of urinary BMA [OR (95% CI) = 0.619 (0.430, 0.889)] decreased the risk of a high level of low-density lipoprotein cholesterol (LDL-C). Higher urinary ttMA levels were positively associated with the ratio of triglycerides to high-density lipoproteins [Q4 compared to Q1: β = 0.11, 95% CI: (0.02, 0.20)]. Higher urinary metabolite levels were negatively associated with the ratio of low-density lipoprotein to high-density lipoprotein [Q4 of ttMA compared to reference: β = -0.06, 95% CI: (-0.11, -0.01); Q4 of BMA compared to reference: β = -0.13, 95% CI: (-0.19, -0.08)].

**Conclusion:**

Benzene and toluene metabolites were significantly and positively associated with hypertriglyceridemia. However, urinary ttMA and BMA levels were negatively associated with high LDL-C levels. These findings suggest that environmental exposure to benzene and toluene disrupts lipid metabolism in humans.

**Supplementary Information:**

The online version contains supplementary material available at 10.1186/s12889-022-14319-x.

## Introduction

Benzene and toluene are pollutants present in the atmosphere [1, 2]. Individuals are unwittingly exposed to benzene and toluene by breathing in outdoor and indoor air [3–5]. These pollutants can also be absorbed into the human body via dermal contact or oral routes [2, 3]. Exposure can be either occupational or environmental [5]. Environmental exposure is more common among the public, and occurs at lower concentrations than occupational exposure. In particular, workers in petrochemical, coke oven, rubber, painting, printing, transportation, and plastic manufacturing industries are easily exposed to high levels of benzene or toluene [2, 6].

The adverse health effects of benzene and toluene on humans have been well-documented over the past few decades. Benzene was designated as ‘group 1, carcinogenic to humans’ by the International Agency for Research on Cancer [7], and can cause various hematopoietic diseases, including myelodysplastic syndrome, acute non-lymphocytic leukemia, chronic lymphocytic leukemia, multiple myeloma, and non-Hodgkin lymphoma [8]. Acute exposure to toluene can lead to severe liver and kidney damage and permanent dysfunction of the central nervous system [2, 9]. However, to date, there has been little discussion of whether exposure to benzene or toluene causes metabolic diseases.

Several epidemiological studies have demonstrated a relationship between environmental exposure to benzene and metabolic diseases [10–15]. In a retrospective cohort study, participants with a high Framingham risk score had significantly higher levels of urinary trans,trans-muconic acid (ttMA), which is a benzene metabolite [10]. Cross-sectional studies have reported that urinary ttMA is associated with metabolic syndrome, oxidative stress, and insulin resistance in children and elderly adults [11–13]. Moreover, a relationship between urinary ttMA and an increased risk of diabetes mellitus (DM) has been found among the adult population of Korea [14, 15]. In the same study, no significant relationship was found between DM and urinary benzylmercapturic acid (BMA), a metabolite of toluene [14]. However, to the best of our knowledge, no studies have investigated whether exposure to benzene and toluene affects the blood lipid profile in humans.

The main aim of this study was to investigate the association between blood lipid levels and urinary ttMA and BMA levels in Korean adults. Additionally, we determined whether environmental exposure to benzene and toluene affected insulin resistance and the risk of cardiovascular disease (CVD). In this study, we used urinary ttMA as an indicator of benzene exposure, and urinary BMA as an indicator of toluene exposure. Urinary ttMA is a useful biomarker for evaluating environmental exposure to benzene at concentrations below 0.1 [16, 17]. Urinary BMA is a valid indicator of human exposure to toluene [18, 19]. In fact, urinary ttMA and BMA are used to evaluate exposure to benzene and toluene in national biomonitoring programs conducted in several countries, including the United States, Canada, and Republic of Korea [20–22].

## Methods

### Study population

This study used cross-sectional data from the Korean National Environmental Health Survey (KoNEHS) Cycle 3 (2015–2017). This nationwide survey provides basic information for monitoring human exposure to environmental chemicals and investigating influential factors. The KoNEHS includes information from interviews, self-report questionnaires, physical examinations, and collection of biological samples. The KoNEHS uses a complex survey design stratified by residential houses, coastal regions, age, sex, and socioeconomic status. The survey was approved by the Institutional Review Board (IRB) of the National Institute of Environmental Research (NIER), Korea (IRB No. NIER-2016-Br-003-01).

A total of 3,787 participants (1,648 males and 2,139 females) aged ≥ 19 years were enrolled in the survey. Among them, we excluded 11 participants with missing data on the urinary metabolites of benzene or toluene, 41 with missing data on lipid profiles, and 312 taking dyslipidemia medications. Finally, 3,423 participants (1,533 males and 1,890 females) were included in the analysis. The ethics review for this analysis was conducted by the IRB of Kyung Hee University Hospital (IRB No. KHUH 2021-08-002). The IRB waived the requirement for informed consent because the study was retrospective.

### Serum lipid profiles

Serum lipid profiles were collected and analyzed according to the KoNEHS guidelines [23]. Total cholesterol (TC) was analyzed using colorimetric analysis (colorimetry, enzymatic method, ADVIA 1800, Siemens) at 505/694 nm. High-density lipoprotein cholesterol (HDL-C) was analyzed by colorimetry (elimination/catalase method, ADVIA 1800, Siemens) after quinonimine was produced using hydrogen peroxide. Triglyceride (TG) was measured for glycerol after hydrolysis with lipoprotein lipase using colorimetry (GPO Trinder without serum blank method, ADVIA 1800, Siemens) [23]. When TG levels were less than 400 mg/dL, low-density lipoprotein cholesterol (LDL-C) levels were measured using the Friedewald formula [24]. Participants with TG levels > 400 mg/dL were excluded from the LDL-C analyses. According to the criteria established by the National Cholesterol Education Program  [25], hypercholesterolemia was defined as TC levels ≥ 240 mg/dL; hypertriglyceridemia was defined as TG levels ≥ 200 mg/dL; low HDL-C levels were defined as < 40 mg/dL in men and < 50 mg/dL in women; and high LDL-C levels were defined as ≥ 130 mg/dL.

We calculated the TG to HDL-C (TG/HDL-C) ratio and LDL-C to HDL-C (LDL-C/HDL-C) ratio. The TG/HDL-C ratio was associated with insulin resistance and helpful in estimating the risk of DM in clinical practice [26–28]. The LDL-C/HDL-C ratio is an indicator of lipid profile imbalance and is used as an indicator of CVD risk [29].

### Measurement of urinary metabolites

Urine samples were collected from sterile cups and transferred to light-blocked storage containers. The container was then transferred to the laboratory in a refrigerated state (2–6 °C) [23]. Urine samples were frozen at -20 °C until analysis [30]. Urinary metabolite concentrations were quantified using high-performance liquid chromatography and mass spectrometry (Agilent 6420 Triple Quadrupole LC-MS) [30]. This method removes unnecessary impurities by passing a solid-phase extraction, eluting the target material and injecting it into a liquid chromatography/mass spectrometer to analyze the sample concentration values using a standard addition method. The C18 (3.5 μm, 2.1 × 100 mm) column was used for chromatography [30]. The mobile phase was prepared by mixing 0.1% acetic acid solution (distilled water): 0.1% acetic acid solution (methanol) in a ratio of 95:5, and the flow rate was 0.3 mL/min [30]. The ionization method for the mass spectrometer was electrospray ionization [30].

Standard solutions were prepared for the range that included the lowest and highest concentrations in the general population. Calibration curves were constructed by adding standard solutions of ttMA at concentrations of 0, 10, 25, 50, 100, 200, 300, and 500 µg/L [30]. Similarly, standard solutions of BMA at concentrations of 0, 0.5, 2, 5, 10, 15, 30, and 50 µg/L were used [30]. The determination coefficient (*R*^2^) of the curves was 0.995 or higher [30]. To maintain the sensitivity of the device, the standard solution was measured after calibration of each of the 20 samples, and the accuracy was measured within ± 15% of the reference value. The limits of detection of ttMA and BMA were 2.3 and 0.197 µg/L, respectively [22]. After adjusting for urine creatinine levels, urinary ttMA concentrations were measured in this study.

Urine creatinine level was determined by measuring the absorbance of picric acid-creatinine complex at 505/571 nm [23]. The picric acid-creatinine complex is formed by the chemical reaction of creatinine with picric acid in an alkaline medium, which is called the Jaffe’s reaction [31]. The ADVIA 1800 (Siemens) was used for creatinine measurements [23].

### Statistical analyses

We conducted an analysis of covariance and the Rao-Scott chi-square test to compare the differences among the study participants concerning the quartiles of urinary ttMA and BMA concentrations. We used a survey-weighted multivariate logistic regression model to calculate the odds ratios (OR) and 95% confidence intervals (CI) for dyslipidemia based on the quartiles of urinary ttMA and BMA. The relationship between the lipoprotein ratios and urinary metabolites of benzene and toluene was examined using multivariate linear regression models. We utilized log-transformed values of the TG/HDL-C and LDL-C/HDL-C ratios because the distribution of each variable was not normal. All multivariate regression models were adjusted for covariates, including age, body mass index (BMI), smoking (never smoker, former smoker, and current smoker), alcohol consumption (never drinker or drinker), exercise (no, low intensity to avoid sweat during exercise, and moderate-intensity as sweat during exercise), educational level (none, less than high school graduation, and more than college), household income (< 871 US dollars, $871–2614, $2614–4357, ≥ 4357 US dollars, and unknown), and marital status (single, married, and others). All statistical analyses were performed using IBM SPSS version 19 for Windows (IBM Corp., Armonk, NY, USA), and stratified variables and weights were applied. Statistical significance was set at *P* < 0.05.

## Results

### Baseline characteristics of the study population

The baseline characteristics of the study population are shown in Table 1. This study included 1,533 (44.79%) men and 1,890 (55.21%) women. The mean concentrations of urinary ttMA were 148.83 (± 5.62) µg/g creatinine in men and 177.86 (± 12.70) µg/g creatinine in women. The mean concentrations of urinary BMA were 7.26 (± 0.47) µg/g creatinine in men and 17.53 (± 6.88) µg/g creatinine in women. The concentrations of urinary ttMA and BMA were significantly higher in women than in men. There was no significant difference between serum TC and LDL-C levels among men and women; however, serum TG level, TG/HDL-C ratio, and LDL-C/HDL-C ratio were higher in men, and serum HDL-C levels were higher in women (*p* < 0.001).

**Table 1:** Baseline characteristics of the study population

Characteristics		Total n = 3423	Males n = 1533	Females n = 1890	*p* value
Age (years)		45.88 (± 0.52)	45.23 (± 0.53)	46.55 (± 0.82)	0.034
BMI (kg/m2)		24.26 (± 0.10)	24.87 (± 0.13)	23.64 (± 0.12)	< 0.001
Education, n (%)	None	106 (3.10)	16 (1.04)	90 (4.76)	0.708
	≤ High school	2056 (60.06)	878 (57.27)	1178 (62.33)	
	≥ College	1261 (36.84)	639 (41.68)	622 (32.91)	
Marital status, n (%)	Single	408 (11.92)	235 (15.33)	173 (9.15)	< 0.001
	Married, cohabited	2653 (77.51)	1218 (79.45)	1435 (75.93)	
	Other (divorce, separation)	362 (10.58)	80 (5.22)	282 (14.92)	
Household income (US dollars), n (%)	< 871	610 (17.82)	245 (15.98)	365 (19.31)	0.012
	871–2614	1366 (39.91)	639 (41.68)	727 (48.47)	
	2614–4357	876 (25.59)	395 (25.77)	481 (25.45)	
	≥ 4357	556 (16.24)	249 (16.24)	307 (16.24)	
	Unknown	15 (0.44)	5 (0.33)	10 (0.53)	
Smoking, n (%)	Never	2166 (63.28)	372 (24.27)	1794 (94.92)	< 0.001
	Former	689 (20.13)	649 (42.33)	40 (2.12)	
	Current	568 (16.59)	512 (33.40)	56 (2.96)	
Alcohol, n (%)	Never drinker	658 (19.22)	127 (8.28)	531 (28.10)	< 0.001
	Drinker	2765 (80.78)	1406 (91.72)	1359 (71.90)	
Exercise, n (%)	No	901 (26.32)	801 (52.25)	1100 (58.20)	0.002
	Low intensity	266 (7.77)	122 (7.96)	144 (7.62)	
	Moderate intensity	1266 (36.99)	610 (39.79)	646 (34.18)	
Urinary metabolites (µg/g⋅creatinine)	ttMA	163.11 (± 7.38)	148.83 (± 5.62)	177.86 (± 12.70)	0.025
	BMA	12.31 (± 3.44)	7.26 (± 0.47)	17.53 (± 6.88)	0.136
Blood lipid levels	TC (mg/dL)	186.10 (± 0.87)	185.87 (± 1.49)	186.33 (± 0.99)	0.804
	TG (mg/dL)	170.27 (± 3.35)	198.55 (± 4.98)	141.06 (± 2.76)	< 0.001
	HDL-C (mg/dL)	56.14 (± 0.43)	51.57 (± 0.50)	60.86 (± 0.52)	< 0.001
	^a^LDL-C (mg/dL)	98.12 (± 0.83)	98.31 (± 1.47)	97.93 (± 0.88)	0.830
	TG/HDL-C ratio	3.51 (± 0.09)	4.33 (± 0.13)	2.66 (± 0.07)	< 0.001
	^a^LDL-C/HDL-C ratio	1.83 (± 0.02)	1.97 (± 0.04)	1.70 (± 0.02)	< 0.001
The continuous variables are presented as mean ± (standard deviation), and the categorical variables are presented as n (%). Urinary metabolites levels were presented after creatinine adjustment. ^a^LDL was calculated by the Friedewald formula after excluding persons with TG > 400 (mg/dL). BMI, body mass index; ttMA, trans,trans-muconic acid; BMA, benzylmercapturic acid; TC, total cholesterol; TG, triglyceride; HDL-C, high-density lipoprotein; LDL-C, low-density lipoprotein					

### Association between blood lipid profiles and urinary metabolites of benzene and toluene

Multivariate logistic regression analysis was conducted to estimate the association between dyslipidemia and the urinary metabolites of benzene and toluene (Table 2). Compared with the reference quartile of urinary ttMA, the adjusted OR for hypertriglyceridemia in second, third and fourth quartiles were 1.433 (95% CI, 1.107–1.856), 1.397 (95% CI, 1.037–1.881) and 1.599 (95% CI, 1.231–2.077), respectively. Compared with the reference quartile of urinary ttMA, the OR for high levels of LDL-C decreased to 0.681 (95% CI, 0.475–0.976) in the third quartile and 0.654 (95% CI, 0.446–0.961) in the fourth quartile. For urinary BMA, the OR for hypertriglyceridemia were 1.486 (95% CI, 1.105–1.998), 1.579 (95% CI, 1.129–2.208) after adjusting for all covariates in the second and third quartiles, respectively. Compared with the reference quartile of urinary BMA, the adjusted OR for high LDL-C levels decreased only in the third quartile to 0.619 (95% CI, 0.430–0.889).

Multivariate linear regression was performed to assess the linear association between serum lipid profiles and urinary metabolites of benzene and toluene (Table 3). Urinary ttMA levels were positively associated with serum TG levels in the second and fourth quartiles after covariate adjustment (The second quartile (Q2) compared to Q1: β = 0.08, 95% CI: [0.01, 0.15], Q4 compared to Q1: β = 0.13, 95% CI: [0.06, 0.20]). Both urinary ttMA (Q4 compared to Q1: β = -0.06, 95% CI: [-0.10, -0.02]) and urinary BMA (Q4 compared to Q1: β = -0.10, 95% CI: [-0.14, -0.05]) levels were negatively associated with serum LDL-C levels.

**Table 2:** Odds ratio and 95% confidence intervals for dyslipidemia according to quartiles of urinary metabolites of benzene and toluene among Korean adults (n = 3423)

	Hypercholesterolemia (≥ 240 mg/dL)		Hypertriglyceridemia (≥ 200 mg/dL)		Low level of HDL-C (< 40 mg/dL for males and < 50 mg/dL for females)		^a^High level of LDL-C (≥ 130 mg/dL)	
	**Unadjusted**	**Adjusted**	**Unadjusted**	**Adjusted**	**Unadjusted**	**Adjusted**	**Unadjusted**	**Adjusted**
Urinary ttMA								
Q1	Ref.	Ref.	Ref.	Ref.	Ref.	Ref.	Ref.	Ref.
Q2	1.269 (0.805–2.001)	1.194 (0.745–1.919)	1.610 (1.233–2.101)	1.433 (1.107–1.856)	1.093 (0.821–1.455)	1.047 (0.771–1.422)	0.827 (0.568–1.204)	0.774 (0.528–1.134)
Q3	0.857 (0.546–1.345)	0.867 (0.556–1.353)	1.547 (1.164–2.055)	1.397 (1.037–1.881)	0.852 (0.641–1.133)	0.813 (0.605–1.092)	0.692 (0.485–0.987)	0.681 (0.475–0.976)
Q4	0.886 (0.580–1.403)	0.878 (0.545–1.417)	1.750 (1.383–2.213)	1.599 (1.231–2.077)	1.035 (0.758–1.414)	1.069 (0.771–1.482)	0.637 (0.439–0.925)	0.654 (0.446–0.961)
*p* for trend	0.158	0.215	0.055	0.087	0.676	0.953	0.017	0.053
Urinary BMA								
Q1	Ref.	Ref.	Ref.	Ref.	Ref.	Ref.	Ref.	Ref.
Q2	1.025 (0.654–1.607)	1.013 (0.657–1.561)	1.128 (0.857–1.484)	1.486 (1.105–1.998)	1.065 (0.796–1.425)	0.906 (0.678–1.212)	0.841 (0.612–1.155)	0.806 (0.581–1.119)
Q3	0.817 (0.499–1.336)	0.753 (0.460–1.230)	1.195 (0.891–1.604)	1.579 (1.129–2.208)	1.124 (0.835–1.514)	0.780 (0.589–1.033)	0.736 (0.516–1.050)	0.619 (0.430–0.889)
Q4	0.809 (0.490–1.334)	0.770 (0.459–1.294)	0.989 (0.722–1.353)	1.338 (0.946–1.894)	1.258 (0.962–1.646)	0.812 (0.623–1.058)	0.785 (0.533–1.154)	0.668 (0.440–1.015)
*p* for trend	0.241	0.192	0.559	0.821	0.088	0.180	0.398	0.109
^a^Participants with TG levels > 400 mg/dL were excluded from LDL-C analysis (n = 3230). Adjusted model was adjusted for age, sex, BMI, education level, marital status, household income level, smoking, alcohol consumption, and exercise. ttMA, trans,trans-muconic acid; BMA, benzylmercapturic acid; HDL-C, high-density lipoprotein; LDL-C, low-density lipoprotein; TG: triglyceride								

**Table 3:** β and 95% confidence intervals for lipid profiles according to quartiles of urinary metabolites of benzene and toluene in the Korean adult (n = 3423)

	**Total cholesterol**		**Triglyceride**		**HDL-C**		^**a**^ **LDL-C**	
	**Unadjusted**	**Adjusted**	**Unadjusted**	**Adjusted**	**Unadjusted**	**Adjusted**	**Unadjusted**	**Adjusted**
Urinary ttMA								
Q1	Ref.	Ref.	Ref.	Ref.	Ref.	Ref.	Ref.	Ref.
Q2	0.00 (-0.02, 0.03)	-0.01 (-0.03, 0.02)	0.14 (0.05, 0.23)	0.08 (0.01, 0.15)	-0.03 (-0.06, 0.01)	-0.01 (-0.04, 0.02)	-0.03 (-0.07, 0.01)	-0.05 (-0.08, -0.01)
Q3	-0.01 (-0.03, 0.01)	-0.01 (-0.04, 0.01)	0.10 (0.01, 0.18)	0.04 (-0.03, 0.11)	0.01 (-0.02, 0.05)	0.02 (-0.01, 0.05)	-0.06 (-0.10, -0.02)	-0.06 (-0.10, -0.03)
Q4	0.00 (-0.02, 0.02)	-0.01 (-0.03, 0.02)	0.19 (0.12, 0.27)	0.13 (0.06, 0.20)	-0.01 (-0.04, 0.03)	-0.01 (-0.04, 0.03)	-0.05 (-0.09, -0.02)	-0.06 (-0.10, -0.02)
*p* for trend	0.932	0.979	0.225	0.159	0.186	0.853	0.325	< 0.001
Urinary BMA								
Q1	Ref.	Ref.	Ref.	Ref.	Ref.	Ref.	Ref.	Ref.
Q2	-0.01 (-0.03, 0.01)	-0.01 (-0.03, 0.01)	-0.01 (-0.08, 0.07)	0.06 (-0.01, 0.12)	0.05 (0.01, 0.08)	0.02 (-0.01, 0.05)	-0.03 (-0.07, 0.01)	-0.04 (-0.08, -0.01)
Q3	0.00 (-0.02, 0.02)	-0.02 (-0.04, -0.01)	0.01 (-0.06, 0.09)	0.06 (-0.01, 0.11)	0.06 (0.03, 0.10)	0.04 (0.01, 0.07)	-0.05 (-0.09, -0.01)	-0.09 (-0.13, -0.05)
Q4	-0.03 (-0.05, -0.01)	-0.04 (-0.06, -0.01)	-0.02 (-0.11, 0.06)	0.03 (-0.03, 0.10)	0.05 (0.01, 0.08)	0.02 (-0.01, 0.06)	-0.07 (-0.12, -0.02)	-0.10 (-0.14, -0.05)
*p* for trend	0.055	< 0.001	0.684	0.361	0.989	0.052	0.115	< 0.001
^a^Participants with TG levels > 400 mg/dL were excluded from LDL-C analysis (n = 3230). Adjusted model was adjusted for age, sex, BMI, education level, marital status, household income level, smoking, alcohol consumption and exercise. ttMA, trans,trans-muconic acid; BMA, benzylmercapturic acid; HDL-C, high-density lipoprotein; LDL-C, low-density lipoprotein								

### Association between lipoprotein ratio and urinary metabolites of benzene and toluene

The highest quartile of urinary ttMA levels was positively associated with a 0.11 [95% CI (0.02, 0.20)] increase in TG/HDL-C ratio (Table 4). In contrast, higher ttMA levels were negatively associated with the LDL-C/HDL-C ratio in the study population after the covariate adjustment, and the β of Q4 compared to Q1 was − 0.06 [95% CI (-0.11, -0.01)]. Urinary BMA levels were also negatively associated with LDL-C/HDL-C ratio in the overall population after the covariate adjustment, in which the β of Q4 compared to Q1 was − 0.13 [95% CI (-0.19, -0.08)].

**Table 4:** β and 95% confidence intervals for lipoprotein ratio according to quartiles of urinary metabolites of benzene and toluene among Korean adults (n = 3423)

	**TG/HDL-C ratio**		^**a**^ **LDL-C/HDL-C ratio**	
	**Unadjusted**	**Adjusted**	**Unadjusted**	**Adjusted**
Urinary ttMA				
Q1	Ref.	Ref.	Ref.	Ref.
Q2	0.13 (0.03, 0.24)	0.07 (-0.01, 0.16)	-0.01 (-0.06, 0.04)	-0.03 (-0.08, 0.01)
Q3	0.07 (-0.04, 0.17)	0.03 (-0.06, 0.12)	-0.07 (-0.12, -0.02)	-0.09 (-0.13, -0.04)
Q4	0.11 (0.01, 0.21)	0.11 (0.02, 0.20)	-0.07 (-0.12, -0.01)	-0.06 (-0.11, -0.01)
*p* for trend	0.093	0.044	0.002	0.003
Urinary BMA				
Q1	Ref.	Ref.	Ref.	Ref.
Q2	-0.05 (-0.15, 0.04)	0.04 (-0.03, 0.01)	-0.08 (-0.14, -0.03)	-0.07 (-0.12, -0.02)
Q3	-0.05 (-0.15, 0.04)	0.02 (-0.05, 0.12)	-0.12 (-0.17, -0.07)	-0.14 (-0.19, -0.09)
Q4	-0.07 (-0.18, 0.04)	0.01 (-0.08, 0.09)	-0.12 (-0.18, -0.06)	-0.13 (-0.19, -0.08)
*p* for trend	0.216	0.999	< 0.001	< 0.001
^a^Participants with TG levels > 400 mg/dL were excluded from LDL-C analysis (n = 3230). Adjusted model was adjusted for age, sex, BMI, education level, marital status, household income level, smoking, alcohol consumption and exercise. ttMA, trans,trans-muconic acid; BMA, benzylmercapturic acid; TG, triglyceride; HDL-C, high-density lipoprotein; LDL-C, low-density lipoprotein				

## Discussion

In this study, we observed a relationship between lipid profiles and urinary metabolites of benzene and toluene in Korean adults. Urinary ttMA and BMA levels were associated with an increased risk of hypertriglyceridemia. In contrast, both urinary ttMA and BMA levels were found to be negatively correlated with serum LDL-C levels. Regarding the relationship between lipoprotein ratio and urinary ttMA and BMA, urinary ttMA was positively associated with the TG/HDL ratio, and both metabolites were inversely related to the LDL-C/HDL-C ratio.

Urinary ttMA was positively associated with serum TG levels and negatively associated with serum LDL-C levels. These findings on the associations between urinary ttMA and blood lipid levels are different from those of previous animal studies [10, 32]. Mice that inhaled volatile benzene had increased levels of serum LDL-C, TC, and HDL-C [10]. For orally administered benzene in mice, the plasma TC level decreased in proportion to the exposure dose, and there were no significant changes in blood TG, HDL-C, and LDL-C levels [32]. This discrepancy may be due to differences in benzene metabolism, depending on concentration and species. Benzene can have different effects on animals and humans because of quantitative differences in the fraction of metabolic pathways [33]. Mice metabolize more hydroquinone metabolites than primates [33]. Additionally, the metabolism of benzene differs between high and low exposure concentrations [34]. In previous animal studies, the concentration of benzene in mice was significantly higher than that in humans [10, 32]. In contrast, the association between urinary BMA and hypertriglyceridemia was in accordance with previous research. Rabbits exposed to a dose of toluene (0.5 mg/kg) have been reported to develop hypertriglyceridemia and glucose intolerance [35].

The effects of benzene exposure on blood lipid profiles can be explained by molecular biological mechanisms. A metabolomic study in humans reported that metabolic pathways, including carnitine shuttle, fatty acid metabolism, glycolysis, and gluconeogenesis were increased in workers exposed to benzene [36]. Benzene induced the expression of enzymes involved in the beta-oxidation pathway and fatty acid transfer in the mitochondria of male C3H/He mice [37]. Recently, Cui et al. reported that crucial genes involved in lipid metabolism, including peroxisome proliferator-activated nuclear receptor gamma, are downregulated in mice exposed to benzene [32]. Additionally, the mRNA expression of adiponectin and leptin was significantly decreased in benzene-exposed white adipose tissues [32]. Changes in the transcription of genes involved in energy metabolism at the molecular level may affect the blood lipid profile in humans [38, 39]. However, the effects of toluene on the expression of genes involved in metabolic pathways have not been studied.

The relationship between TG/HDL-C ratio, an indicator of insulin resistance, and urinary ttMA levels revealed in this study is in line with previous researches [12–15]. An association between urinary ttMA levels and insulin resistance has been reported in children, adolescents, and elderly adults [12, 13]. Additionally, several studies have revealed that benzene metabolites are associated with an increased risk of DM [14, 15]. During benzene metabolism, Cytochrome P450 (CYP) 2E1 produces reactive oxygen species and free radicals, leading to oxidative stress [40–42]. Oxidative stress plays a role in the development of insulin resistance by interrupting insulin signaling pathways and dysregulating adipocytokines [43, 44]. In an animal study, C57B/6 mice exposed to benzene showed insulin resistance by inhibiting insulin-stimulated Akt phosphorylation and enhanced nuclear kappa phosphorylation [45]. Treatment with TEMPOL, a superoxide dismutase mimetic, restores this alteration, demonstrating that benzene-induced oxidative stress enhances insulin resistance [45]. Moreover, exposure of pregnant C57BL/6JB mice to benzene resulted in glucose intolerance and severe insulin resistance in male offspring [46].

Insulin resistance and hypertriglyceridemia have been studied in depth, and a complicated relationship has been identified. White adipose tissue releases free fatty acids (FFA) into the blood when the body is insulin-resistant, and skeletal muscle cells and the liver obtain increased amounts of FFA [47, 48]. Excessive influx of FFA into skeletal muscle cells promotes the accumulation of ceramide and diacylglycerols, which inhibit the translocation of glucose transporter type 4 and the Akt/PKB signaling pathway [49]. Free fatty acid promotes the formation of very low-density lipoprotein with high TG concentration in the liver, resulting in hypertriglyceridemia [50]. Hypertriglyceridemia causes lipotoxicity by accumulating fatty acids in other tissues, which worsens systemic insulin resistance [51]. Therefore, the findings of this paper, which revealed the positive association between urinary metabolites of benzene and toluene, hypertriglyceridemia, and insulin resistance, point in the same direction.

Therefore, LDL-C/HDL-C is a well-known risk indicator of CVD and the progression of atherosclerosis [29, 52, 53]. Hypercholesterolemia and low HDL-C levels are critical contributors of CVD development [54, 55]. In this study, increased concentrations of urinary ttMA and BMA were observed to have a strong relationship with decreased LDL-C and LDL-C/HDL-C levels. It is necessary to confirm whether the anti-atherogenic effects of benzene and toluene have been reproduced in other population studies.

### Strengths and limitations

To our knowledge, this is the first study to explore the association between lipid profiles and exposure to benzene and toluene in a general population. The potential lipid metabolism-disrupting effects of benzene and toluene are supported by the mechanisms revealed in animal experiments. However, this study has several limitations. First, as this was a cross-sectional study, the findings can only be used to indicate associations and not to assess causal relationships. Second, the lifestyle behaviors and medical history of the participants were investigated through interviews and questionnaires. Self-reporting may lead to recall bias and incorrect classification [56]. Third, this study did not consider individual-specific gene expression or polymorphisms. Individual sensitivity to benzene exposure may be influenced by nucleotide polymorphisms in NQO1, MPO, CYP2E1, GSTT1, and GSTM1 [57, 58]. Genetic polymorphisms in ALDH2, CYP1A1, CYP2E1, GSTM1, and GSTT2 can affect their ability to metabolize toluene [59, 60]. Fourth, the specificity of ttMA in assessing environmental exposure to benzene may have some limitations. It has been reported that ttMA levels were not correlated with actual exposure to benzene at exposure levels below 0.5 ppm [61]. Moreover, urinary ttMA levels are affected by individual sorbic acid intake [62]. Trans,trans-muconic acid is a metabolite of sorbic acid that is commonly used as a preservative in a wide range of food [63]. Fifth, our findings on serum LDL-C levels may be limited by the inaccuracy of the Friedewald formula. In patients with moderate to high LDL-C levels, Friedewald estimation yields an accurate result [64, 65]. However, the Friedewald equation is unreliable to calculate serum LDL-C levels in patients with low LDL-C (< 70 mg/dL) [64, 65]. In this study, 534 (16.53%) participants had LDL-C levels < 70 mg/dL (Supplementary Fig. 1).

## Conclusion

This cross-sectional study suggest that human lipid metabolism may be altered by exposure to benzene and toluene. The urinary metabolites of benzene and toluene are associated with an increased risk of hypertriglyceridemia. Additionally, TG/HDL-C levels increased in individuals with high urinary ttMA levels. The urinary metabolites of benzene and toluene were negatively associated with serum LDL-C levels. Further studies in other ethnic groups are required to verify these findings.

## Electronic supplementary material

Below is the link to the electronic supplementary material.

Supplementary Material 1

## Data Availability

The database used in the present study can be used after requesting the KoNEHS data from the National Institute of Environmental Research in Korea.

## List of abbreviations

ttMA: Trans,trans-muconic acid;
BMA: Benzylmercapturic acid;
KoNEHS: Korean National Environmental Health Survey;
CVD: Cardiovascular disease;
TC: Total cholesterol;
TG: Triglyceride;
LDL-C: Low-density lipoprotein cholesterol;
HDL-C: High-density lipoprotein cholesterol;
TG/HDL-C: Ratio of triglyceride to high-density lipoprotein cholesterol;
LDL-C/HDL-C: Ratio of low-density lipoprotein to high-density lipoprotein cholesterol;
DM: Diabetes mellitus;
CYP: Cytochrome P450;
IRB: Institutionall Review Board;
NIER: National Institute of Environmental Research;
BMI: Body mass index;
OR: Odds ratio;
CI: Confidence interval;
FFA: Free fatty acid.
