# Supplementary material for: Association of metabolites of benzene and toluene with lipid profiles in Korean adults: Korean National Environmental Health Survey (2015–2017)
Source: BMC Public Health. 2022 Oct 14;22:1917. doi: 10.1186/s12889-022-14319-x (PMC9569087; doi:10.1186/s12889-022-14319-x)
Supplement: Supplementary file 1 — Supplementary Material 1 [file 12889_2022_14319_MOESM1_ESM.ppt]

## Slide 1
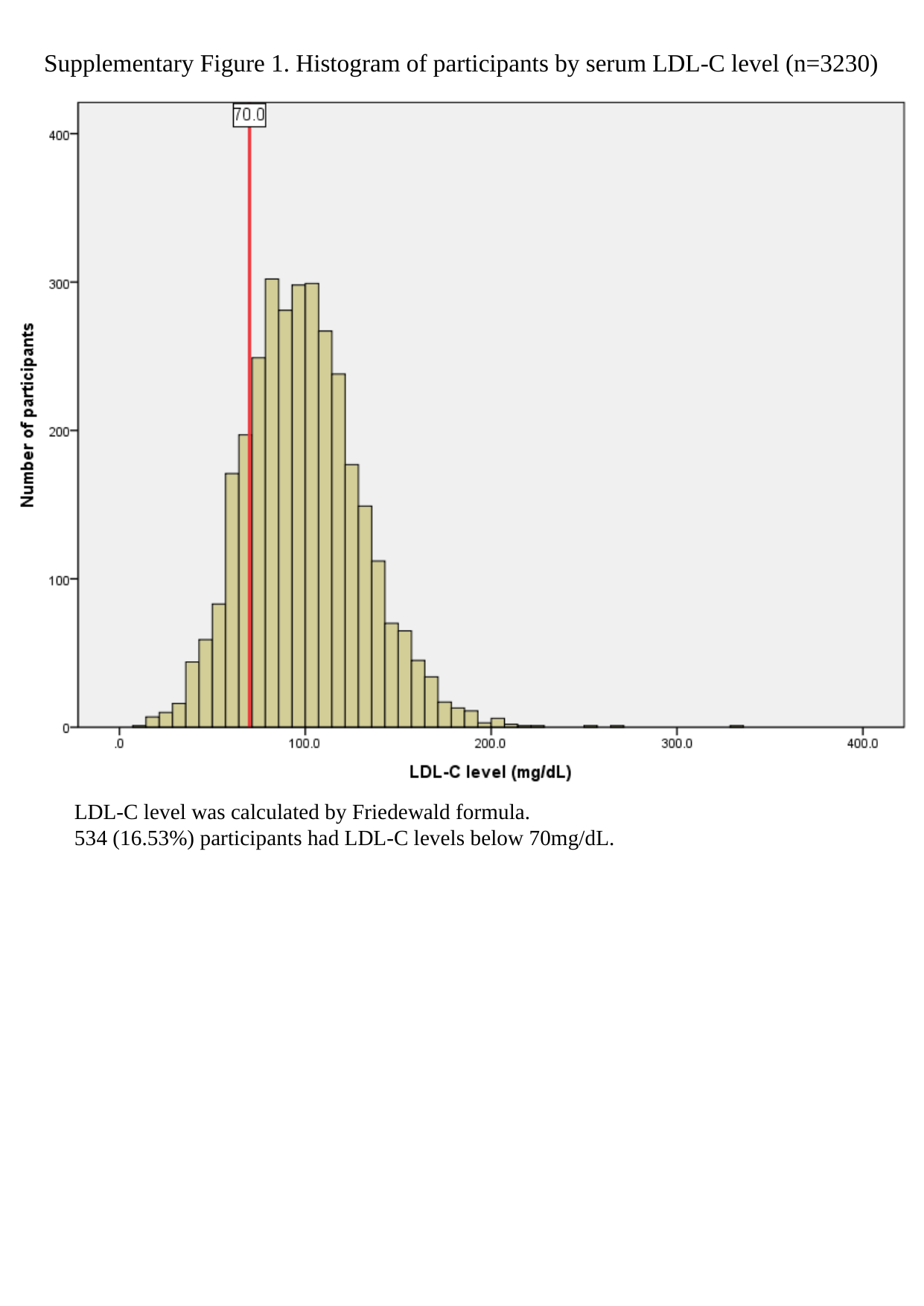

Supplementary Figure 1. Histogram of participants by serum LDL-C level (n=3230)
LDL-C level was calculated by Friedewald formula.
534 (16.53%) participants had LDL-C levels below 70mg/dL.
